# Insights from using an outcomes measurement properties search filter and conducting citation searches to locate psychometric articles of tools used to measure context attributes

**DOI:** 10.1186/s13104-023-06294-2

**Published:** 2023-03-11

**Authors:** Wilmer J. Santos, Alison M. Hutchinson, Tamara Rader, Ian D. Graham, Vanessa Watkins, Ligyana Korki de Candido, Megan Greenough, Janet E. Squires

**Affiliations:** 1 Clinical Epidemiology Program, Ottawa Hospital Research Institute, Ottawa, Canada; 2 School of Nursing and Midwifery, Deakin University, Geelong, Australia; 3 Barwon Health, Geelong, Australia; 4 Canadian Agency for Drugs and Technologies in Health, Ottawa, Canada; 5 Faculty of Health Sciences, School of Nursing, University of Ottawa, Ottawa, Canada; 6 Faculty of Medicine, School of Epidemiology and Public Health, University of Ottawa, Ottawa, Canada

**Keywords:** Psychometric, Context, Filter, Outcomes, Review, Search

## Abstract

**Objectives:**

To describe our experience with using a methodological outcomes measurement search filter (precise and sensitive versions of a filter designed to locate articles that report on psychometric properties of measurement tools) and citation searches to locate psychometric articles for tools that can be used to measure context attributes. To compare the precise filter when used alone and with reference list checking to citation searching according to number of records found, precision, and sensitivity.

**Results:**

Using the precise filter, we located 130 of 150 (86.6%) psychometric articles related to 22 of 31 (71.0%) tools that potentially measured an attribute of context. In a subset of six tools, the precise filter alone was more precise than searching with the precise filter combined with reference list searching, or citation searching alone. The precise filter combined with reference list checking was the most sensitive search method examined. Overall, we found the precise filter helpful for our project as it decreased record screening time. For non-patient reported outcomes tools, we had less success with locating psychometric articles using the precise filter because some psychometric articles were not indexed in PubMed. More research that systematically evaluates database searching methods is needed to validate our findings.

**Supplementary Information:**

The online version contains supplementary material available at 10.1186/s13104-023-06294-2.

## Introduction

Evaluation of context is important for the translation of research into policy and practice [1–4]. Evaluation is the process by which implementation strategies, innovation, or context are tested using valid and reliable tools [5] to determine their ability to influence research uptake or outcomes [6]. Systematic identification of psychometric articles can be resource intensive and time-consuming [7]. Psychometrics are properties of measurement tools that dictate their ability to capture the concept they are intended to measure, minimize measurement error and detect change over time [8] Many validated search filters [9] have been developed to mitigate the workload associated with searching the extensive amount of published literature indexed in bibliographic databases. Methodological search filters are a combination of search terms that ensure the retrieval of a particular type of article [10]. Terwee and colleagues [10] developed a methodological search filter to identify psychometric articles indexed in PubMed for tools that measure patient-reported outcomes (PROM) (tools measuring health outcomes and daily functions reported by patients [10, 11]) and potentially non-PROM measurement tools. This outcome measurement properties (OMP) filter has a sensitive and a precise version, hereafter referred to as the “sensitive filter” or the “precise filter”. The sensitive filter was designed for systematic reviews and the precise filter for gathering psychometric articles without the need for a comprehensive search [10]. It is important to validate search filters to ensure that there is a balance between sensitivity (ability to identify relevant articles) and precision (minimize number of records) [10, 12].

We completed a series of three large international studies to develop the Implementation in CONtext (ICON) framework [1–3]: (1) a concept analysis of context as reported in the international published literature (n = 70 articles) [1]; (2) an analysis of 145 interviews conducted with a wide range of healthcare providers in multiple healthcare settings for elements of context relevant to research use in clinical practice [2]; (3) a qualitative interview study with 39 health system stakeholder (individuals responsible for change in a healthcare organization) in 4 countries to elicit their perceptions of important elements of context for implementation [3]. The findings from these studies were then triangulated to produce ICON, a meta-framework where context is conceptualized in three levels (micro, meso, and macro) that are divided into five domains, 22 core attributes and 108 features [13]. We aim to create a repository of tools that measure ICON attributes, and to summarize the psychometric properties of these tools. However, the heterogeneity in the constructs and populations associated with ICON attributes made it difficult to construct a search strategy, guided by traditional systematic review methods [14, 15], that had a good balance between sensitivity and precision. We used Terwee and colleagues’ [10] filter (both versions) to refine our search for psychometric articles. Since a tool name was required to locate psychometric articles of specific tools, we performed citation searches [16, 17] to locate articles for unnamed tools (Additional File 1: search method definitions).

The purpose of this research note is to describe our experience of using an OMP filter and citation searching to identify psychometric articles for 31 tools [18–48] that measure ICON attributes. Our objectives are to: (1) provide an overview of our experience of using an OMP filter (both versions) and citation searching; (2) compare the precise filter with or without reference checking with citation searching according to the number of records located and time required to complete article selection, precision, and sensitivity; (3) discuss the benefits of and the insights garnered from using an OMP filter and citation searching.

## Methods

In consultation with a health information specialist (TR), we performed searches in PubMed from inception to July/December 2021 using the precise filter combined with the tool’s name (Additional File 2: search terms). We used the sensitive filter only if the search using the precise filter produced zero results. We performed citation searches using an indexed development article when: (1) tools had no name; or (2) the OMP (both versions) filter search did not locate a relevant article. Citation searching was conducted in Scopus and Web of Science. We checked the reference lists of included articles to supplement our database searches [17, 49]. We (LKC, MG, WJS or VW) screened records and full text articles independently and in duplicate. Discrepancies were resolved through consensus or in consultation with a senior researcher. We included articles that aimed to evaluate the psychometric properties of any of the 31 tools [18–48].

Nineteen of 31 (61.3%) tools were non-PROM [22, 24–27, 29–31, 33, 34, 36, 38, 39, 41–44, 46, 47], and the remaining 12 (38.7%) were PROM [18–21, 23, 28, 32, 35, 37, 40, 45, 48]. We conducted a search on PubMed using an OMP filter for 29 of 31 (93.5%) tools which had names; 12 PROM [18–21, 23, 28, 32, 35, 37, 40, 45, 48] and 17 non-PROM [22, 25–27, 29–31, 33, 34, 36, 38, 39, 41–44, 46]. We used the precise filter for 28 of 29 named tools [18–23, 25–35, 37–46, 48], and the sensitive filter for one tool [36] (precise version found zero records). We performed a citation search on 2 of 31 tools (6.5%) (both non-PROM [24, 47]) because they were unnamed.

### Methods for sub-analysis

We compared the search results of the precise filter (alone or combined with reference checking), with citation searching in a sub-analysis of six randomly selected tools (three PROM [28, 35, 40] and three nonPROM [26, 31, 43]). We compared the search methods according to number of records identified, time to complete record screening, precision, and sensitivity. One researcher appraised the inclusion of articles for the citation searches. Precision is the total number of included articles divided by the total number of records identified by a search method [14]. Sensitivity is the number of included articles divided by the total number of unique articles included across the three search methods [14].

## Results

We found at least one relevant article for 27 of 31 (87.1%) tools [20–28, 30–33, 35–48] (Table 1: searches and search results). Across all search methods, we screened 10,303 records, appraised 546 full-text articles for eligibility and included 150 articles (Additional file 3 and Additional file 4: PRISMA diagram and search results for each tool respectively).

**Table 1:** Description of search results according to number of tools

Criteria	[N (%)]	PROM [N (%)]	Non-PROMs [N (%)]
Outcomes measurement properties (both versions) Filter (N = 29 tools)^a^			
*Subtotal*—Number of named tools for which a search using the outcomes measurement properties (both versions) filter was performed	**29/31 (93.5%)**	**12/12 (100.0%)**	**17/19 (89.5%)**
• Number of tools for which a relevant article was not found	7 (24.1%)	2 (16.7%)	5 (41.7%)
• Number of tools for which at least one relevant article was found	22 (75.9%)	10 (83.3%)	12 (70.6%)
Precise Filter^b^—Reference checking on identified relevant articles (N = 22 tools)			
*Subtotal*—Number of tools with relevant articles for which we performed a search of their reference list	**22/31 (71.0%)**	**10/12 (83.3%)**	**12/19 (70.6%)**
• Number of tools for which at least one additional relevant article was found	5 (22.7%)	2 (20.0%)	3 (25%)
• Number of tools for which additional relevant articles found via reference checking are not indexed in PubMed	4/5 (80.0%)	1/2 (50.0%)	3/3 (100%)
• Number of tools for which additional relevant articles found via reference checking are indexed in PubMed	1/5 (20.0%)	1/2 (50.0%)	0/3 (0.0%)
Citation Search (N = 9 tools)			
*Subtotal*—Number of tools for which a citation search was performed	**9/31 (29.0%)**	**2/12 (16.7%)**	**7/19 (36.8%)**
• Number of named tools for which at least one relevant article was found	3 (33.3%)	0 (0.0%)	3 (42.9%)
• Number of named tools for which a citation search of known development article could not be performed on Scopus or Web of Science	3 (33.3%)	2 (100%)	1 (14.3%)
• Number of named tools for which a citation search did not find a relevant article	1 (11.1%)	0 (0.0%)	1 (14.3%)
• Number of unnamed tools for which at least one relevant article was found	2 (22.2%)	0 (0.0%)	2 (28.6%)
Citation Search—reference checking on identified relevant articles (N = 5 tools)^c^			
*Subtotal*—Number of tools with relevant articles for which we performed a search of their reference list	5/31 (16.1%)	0/12 (0.0%)	5/19 (26.3%)
• Number of tools for which at least one additional relevant article was found	0 (0.0%)	0 (0.0%)	0 (0.0%)
Total number of tools^d^	**31**	**12/3 (38.7%)**	**19/31 (61.3%)**

Bold values refer to the total or subtotal number of tools for each search method and/or type of tool (PROM or non-PROM).

^a^The sensitive filter was used for the “Role Based Performance Scale” because the precise filter did not identify any records. ^b^Reference checks could not be performed on four tools (two PROMS (“Art of Medicine survey” [19] and “American Board of Internal Medicine's (ABIM) questionnaire” [18]) and two Non-PROMs (“Questionnaire on Computer Systems and Decision making” [34] and “Leadership Behavior Description Questionnaire” [29])) because there were no included articles.^c^Only the searches for which we used the precise filter resulted in relevant articles. ^d^The subtotals for the searches performed using the OMP filter (both versions) and citation search do not add up to 31 because we performed additional citation searches when the OMP filter (both versions) search did not locate an article

### *OMP filter (N* = *29 named tools) and reference checks (N* = *22 tools)*

Our searches using the precise filter successfully identified 130 relevant articles across 22 of 29 (75.9%) named tools [20–23, 26–28, 30–33, 35, 37, 39–46, 48]. We identified a relevant article for a slightly higher proportion of PROMS (10 of 12; 83.3%) [20, 21, 23, 28, 32, 35, 37, 40, 45, 48] than non-PROMS (12 of 17; 70.6%) [22, 26, 27, 30, 31, 33, 39, 41–44, 46]. Our OMP (both versions) filter search did not locate a relevant article for 7 of 29 tools (24.1%), a larger proportion of non-PROM (5 of 17; 41.7%) [25, 29, 34, 36, 38] than PROM (2 of 12; 16.7%) [18, 19].

By searching the reference lists of included psychometric articles, we found 12 additional psychometric articles [43, 50–60] for 5 (two PROM [21, 45] and three non-PROM [30, 39, 43]) of the 22 (22.7%) tools identified using the precise filter. Ten of the 12 (83.3%) articles (one article [50] for a PROM [45] and nine articles [43, 51–53, 56–60] across three non-PROMs [30, 39, 43]) were not indexed in PubMed. The remaining two articles [54, 55] (for one PROM [21]) were indexed in PubMed but not captured by the precise filter search.

### *Citation searches (N* = *9 tools) and reference checks (N* = *5 tools)*

Citation searches were performed using the index tool development article for 9 of 31 (29.0%) tools (two PROM [18, 19] and seven non-PROM [24, 25, 29, 34, 36, 38, 47]). Citation searching retrieved eight psychometric articles across 5 of 9 (55.6%) [24, 25, 36, 38, 47] tools. We did not locate additional articles from reference checking. We were unable to perform a citation search for 3 of 9 (33.3%) tools (two PROM [18, 19] and one non-PROM [29]) because the development study was in a book or an online report and not indexed on Scopus or Web of Science. With citation searching we did not locate a psychometric article for the non-PROM [34] tool.

### Sub-analysis results

Amongst a subset of six tools, the precise filter alone identified 500 records, while the precise filter search combined with reference checks located 1882 records, and citation searching found 2340 records (Table 2: detailed sub-analysis results). The precise filter search alone was also the most time-efficient method in our sub-analysis. We completed screening of records and full-text articles for the six tools four times faster when using the precise filter alone, compared to citation searches (~ 350.5 min versus 1386 min, respectively). The addition of reference checks to the precise filter search increased the time required for article selection (~ 1079.8 min) but was still more time efficient than citation searching.

**Table 2:** Sub-group Analysis of OMP filter (with or without reference checking) and Citation Search

Tool Name	Search Method	# of unique records identified	# of potentially relevant articles	# of included psychometric articles	Precision^a^ [N (%)]	Sensitivity^b^ [N (%)]
Patient reported outcomes (PROM)						
Interpersonal processes of care (PROM)	Precise filter search and reference checks	392	21	9	9/392 (2.3%)	9/9 (100.0%)
	Precise filter alone	124	11	9	9/124 (7.3%)	9/9 (100.0%)
	Citation search	159	8	5	5/159 (3.1%)	5/9 (55.6%)
Risser patient satisfaction scale/instrument (PROM)	Precise filter search and reference checks	245	17	6	6/245 (2.4%)	6/6 (100.0%)
	Precise filter alone	114	11	6	6/114 (5.3%)	6/6 (100.0%)
	Citation search	278	126	6	6/278 (2.2%)	6/6 (100.0%)
Shared decision-making questionnaire (SDM-Q-9) (PROM**)**	Precise filter search and reference checks	489	59	22	22/489 (4.5%)	22/22 (100.0%)
	Precise filter alone	55	31	22	22/55 (40.0%)	22/22 (100.0%)
	Citation search	414	26	20	20/414 (4.8%)	20/22 (91.0%)
Subtotal for PROM	Precise filter search and reference checks	1126	97	37	31/1126 (2.8%)	Mean (SD) = 100.0% (0.0%)
	Precise filter alone	293	53	37	37/293 (12.6%)	Mean (SD) = 100.0% (0.0%)
	Citation search	851	160	31	31/851 (3.6%)	Mean (SD) = 82.2% (23.5%)
Non-patient reported outcomes (non-PROM)						
Implementation leadership Scale (ILS) (nonPROM)	Precise filter search and reference checks	471	22	9	9/471 (1.9%)	9/9 (100.0%)
	Precise filter alone	185	10	9	9/185 (4.9%)	9/9 (100.0%)
	Citation search	194	22	8	8/194 (4.1%)	8/9 (88.9%)
Multiple-group measurement scale for interprofessional collaboration (nonPROM)	Precise filter search and reference checks	97	3	2	2/97 (2.1%)	2/2 (100.0%)
	Precise filter alone	2	2	2	2/2 (100.0%)	2/2 (100.0%)
	Citation search	69	12	2	2/69 (2.9%)	2/2 (100.0%)
Team climate inventory (TCI) and team climate inventory-short (nonPROM)	Precise filter search and reference checks	118	32	8	8/118 (6.8%)	8/10 (80.0%)
	Precise filter alone	20	16	6	6/20 (30.0%)	6/10 (60.0%)
	Citation search	1226	28	8	8/1226 (0.7%)	8/10 (80.0%)
Subtotal for non-PROM	Precise filter search and reference checks	756	57	19	19/756 (2.5%)	Mean (SD) = 93.3% (11.5%)
	Precise filter alone	207	28	17	17/207 (8.2%)	Mean (SD) = 86.7% (23.1%)
	Citation search	1489	62	18	18/1489 (1.2%)	Mean (SD) = 89.6% (10.0%)
Total	**Precise filter search and reference checks**	**1882**	**154**	**56**	**56/1882 (3.0%)**	**Mean (SD) = 96.7% (8.2%)**
	**Precise filter alone**	**500**	**81**	**54**	**54/500 (10.8%)**	**Mean (SD) = 93.3% (16.3%)**
	**Citation search**	**2340**	**222**	**49**	**49/2340 (2.1%)**	**Mean (SD) = 85.9% (16.6%)**

Bold values refer to the total number of records and articles found, the sensitivity and the precision of each search method inclusive of the six tools assessed in the sub-analysis

^a^We calculated precision by taking the total number of included articles and dividing by the total number of records identified by a search method. ^b^We calculated sensitivity  by taking the number of included articles and dividing by the total number of unique articles included across the three search methods

The precise filter combined with reference checks located the most included articles (n = 56 (96.6%)). The precise filter when combined with reference checking was the most sensitive search method (mean, standard deviation = 96.7%, 8.2%) and the precise filter alone was the most precise search method (precision = 10.8%). The precise filter (alone or combined with reference checks) was more sensitive and precise when searching for psychometric articles for the three PROM [28, 35, 40] than for the three non-PROM [26, 31, 43] tools.

The precise filter in combination with reference checks identified nine articles, across four tools [26, 28, 40, 43]; all these articles were previously unidentified through the citation search (Additional file 5: missed articles and reasons they were missed by one of the methods). One [61] of 9 (11.1%) articles relating to one tool [26] was not identified by the citation search because an article [62] other than the development article was cited. Two of 9 (22.2%) articles [51, 63] relating to two tools [40, 43] were missed by our citation search because they were not indexed as a “cited” article in Scopus or Web of Science even though the authors referenced the article we used for citation searching. Two of 9 (22.2%) articles [64, 65] relating to two tools [28, 43] were not identified by citation searching because they were earlier reports about the tools’ development and were not indexed in either Scopus or Web of Science. Four of 9 (44.4%) articles [66–69] relating to two tools [28, 40] were missed by citation searching because the authors cited a different article reporting on the tool’s development from the one we used in our citation search. Additional citation searches were performed for these two tools [28, 40] using the newly discovered development articles [65, 70].

In summary, the additional searches did not change the precision of citation searching across the six tools (2.1%) but did increase the overall mean sensitivity of citation searching from 93.3% to 94.1% (Additional file 6: sub-analysis results with the additional citation searches considered). In contrast, the citation search captured two articles [71, 72], relating to one tool [43], that were not located by the precise filter search because they were not indexed in PubMed.

## Discussion

Our study provides a greater understanding of the benefits of using the precise filter and conducting citation searches (see Table 3).

**Table 3:** Benefits and insights gained from using the outcomes measurement properties filter and conducting a citation search to locate psychometric articles

Outcomes measurement properties filter [10]	
Benefits	
1.Combined with reference checks, the precise filter was more sensitive than using the precise filter by itself or performing citation searching in our sample of six tools 2.The precise filter was more precise when it is used alone than when it is combined with reference checks or when a citation search is performed in our sample of six tools 3.The precise filter decreased the number of records that we had to screen, and the number of full text articles that we needed to locate and screen—which translates to decrease in time and resources needed to allocate to screening 4.Individuals thinking of using the OMP filter have the option of using either the precise and sensitive OMP filter depending on their goal or the resources they have 5.The OMP (sensitive and precise) filter has been validated and is published in an open access journal 6.The OMP (sensitive and precise) filter is easy to use, and its developers provide clear instructions on how to apply the filter in their development and validation study [10] 7.Although this was not the purpose in our study, the OMP (sensitive and precise) filter can be used to help identify psychometric articles for any tool that measure a particular concept of interest	
Insights	
1.Searching using the OMP (sensitive and precise) filter is limited to PubMed, which means that psychometric articles not indexed in PubMed may not be captured a.Checking the reference list of identified psychometric articles may help locate psychometric articles not indexed in PubMed b.From our experience, the precise filter was more successful at identifying articles for PROM than non-PROM, which could potentially be influenced by some non-PROM being developed in other than healthcare disciplines or fields c.Psychometric articles that are not published in open access journals may not be located through PubMed 2.When using the OMP (sensitive and precise) filter to search for the psychometric articles of particular tools, the search is dependent on knowing the tool name a.Tool names are sometimes composed of very commonly used terms that may result in a large increase in the records retrieved. In these instances, we added double quotation marks at the beginning and at the end of the tool’s name b.Some tool names are accompanied with acronyms. These acronyms were not always unique to the tool that we searched for, and sometimes increased the number of irrelevant records retrieved c.Tool names sometimes may differ slightly depending on the authors or variations in the tool name may occur through time. Searches with the precise filter may need to be iteratively modified if new names are found. For example, for the tool named “Care Transitions Measure”, we needed to add the search term “Care Transition Measure” to the search strategy because we found four articles [78−81] in the reference lists of included articles (these articles were indexed in PubMed and locatable by the precise filter) where the word “transitions” was not pluralized in the tool name. We also found slight nomenclature differences across articles for the “Risser Patient Satisfaction Scale” [82–84], which is sometimes referred to as the “Patient Satisfaction Instrument” [35] or “Patient Satisfaction Index” [85, 86] d.An alternative searching method is required when searching for psychometric articles of tools that do not have an explicit name 3.The OMP (precise and sensitive) filter was developed and validated in 2009. Since 2009, there has been advancement in PubMed and in the field of measurement a.In 2019, a new PubMed interface was launched with updated technology, and included changes in search syntax and translations. For example, PubMed improved automatic term mapping by programing the system to detect and alter search results based on the presence of other regional spelling variation (e.g., British, and American spelling) of a term, singular and plural forms, and synonyms [74] i).When we used the OMP (precise and sensitive) filter on PubMed, the following error message appeared: The following terms were not found in PubMed: Studies, ''addresses'', ''lectures'', cases'', works'', ''congresses' b.In 2018, The COSMIN manual [75, 76] was updated to have a greater emphasis on content validity c.In 2021, Stanick and colleagues [77, 87] published a scale for appraising the pragmatic properties of tools	
Citation Search [17, 49]	
Benefits 1.Our approach at citation searching used broader and more general databases (Scopus and Web of Science) which spans beyond the healthcare literature a The citation searching may identify articles that are not indexed on PubMed and therefore cannot be identified through searching with the OMP (precise and sensitive) filter 2.A citation search can be used to search for the psychometric articles of tools that do not have an explicit name or when the search with OMP (precise and sensitive) filter did not locate a psychometric article 3.A citation search is not susceptible to the terms that makes up the tool’s name or the variability pertaining to the tool’s name that may exist	
Considerations 1.The results of citation searching are dependent on the identified development articles used for the search a.Sometimes, development studies are reported in more than one article. Subsequent psychometric articles may cite one of the articles or may cite both b.Performing a citation searching on all development articles and deduplicating the number of records will increase the sensitivity of the search but would require a preliminary search to find the development articles and would also increase the number of records that needs to be screened 2.The way we performed a citation search is limited to the articles indexed in Scopus and Web of Science a.An article that cites the development article used for searching may be missed by the citation search if it is not indexed as a cited article on Scopus and Web of Science b.An article not indexed in Scopus and Web of Science will not be identified c.Books or online reports reporting on the development of tools cannot be used to perform a citation search on Scopus and Web of Science 3.The citation search may identify more records than by searching using the OMP (precise and sensitive) filter; this is especially true for tools that are used a lot in primary studies to evaluate outcomes, but do not aim to evaluate the psychometric properties of the tool	

### OMP filter

The most important benefit of searching using the precise filter was the decreased time required to screen records. Based on our sub-analysis of six tools, searching with the precise filter alone proved to be the most precise method, and the combination of the precise filter with reference checks was the most sensitive search method. However, before using the OMP filter (both versions), the following should be considered: 1) the OMP filter cannot locate articles that are not indexed in PubMed; 2) the search for psychometric articles of particular tools is dependent on knowing the name of the tool; and 3) the unknown implications of recent changes to the PubMed interface [73, 74] (detailed in Table 3) on the OMP filter.

We found that the search conducted with the precise filter successfully identified more psychometric articles for PROM than non-PROM. However, further systematic research comparing the efficacy of the OMP filter at locating psychometric articles for PROM and non-PROM is needed to confirm our findings. Systematic testing of the OMP filter in the context of recent developments in the field of measurement is an avenue for future research (e.g. how the addition of search terms related to content validity (relevance, comprehensiveness, comprehensibility [75, 76]) and pragmatic properties (usefulness, compatibility, acceptability, etc. [77]) affect sensitivity and specificity).

### Citation searching

Citation searching was helpful in identifying psychometric articles of tools that had not been named or tools for which the search with the OMP filter (both versions) did not locate a psychometric article (the majority being non-PROMs). When planning a citation search to locate psychometric studies, the following should be considered: (1) tool development study might be reported in multiple articles; (2) multiple citation searches on several development articles may be more sensitive but increase the number of retrieved records; (3) citation searching is limited to the articles indexed in the utilized database(s); (4) citation searching may identify more records than the precise filter.

### Limitations

We did not intend to conduct a systematic evaluation of the OMP filter or citation searching. Furthermore, we did not evaluate other search methods that are used in measurement reviews (e.g., a traditional search strategy). Our sub-analysis, comparing the precise filter and citation searching, was based on only six tools. The references we used for citation searching were originally retrieved using the OMP filter and only one researcher screened records retrieved by citation searching. Considering these limitations, our findings may reflect trends in each methods’ search results, precision and sensitivity that require further investigation.

## Conclusion

The precise filter decreased the number of records required for screening for our project. Based on a sample of six tools, searching with the precise filter alone was more precise, sensitive, and efficient than citation searching. More systematic research is required to evaluate the usefulness of OMP filters and other search methods in measurement reviews, especially reviews on tools that are developed/used across multiple disciplines and specialties and address a diverse range of theoretical constructs.

## Supplementary Information

**Additional file 1: **Definition of search methods.

**Additional file 2: **Tool names and search terms combined with terwee filter.

**Additional file 3: **PRISMA diagram of identified records screened and full texts appraised.

**Additional file 4: **Search results breakdown for each tool.

**Additional file 5: **Reasons for discrepancies between the precise outcomes measurement properties filter search and citation sear.

**Additional file 6: **Sub-group analysis of terwee filter (with or without reference checking) and citation search.

## Data Availability

The definition of search methods, search strategy, PRISMA diagram, the search results for each tool, reasons for discrepancies between search methods during the sub-analysis, changes in results with additional citation searches for the sub-analysis are provided as additional files. The datasets used and/or analyzed during the current study are available from the corresponding author on reasonable request.
