# Supplementary material for: Insights from using an outcomes measurement properties search filter and conducting citation searches to locate psychometric articles of tools used to measure context attributes
Source: BMC Res Notes. 2023 Mar 11;16:34. doi: 10.1186/s13104-023-06294-2 (PMC10007786; doi:10.1186/s13104-023-06294-2)
Supplement: Supplementary file 1 — Additional file 1: Definition of search methods. [file 13104_2023_6294_MOESM1_ESM.docx]

**Additional File 1 – Definition of Search Methods**

| **Search Method** | **Definition and Rationale** |
| --- | --- |
| Citation Searching^1,2^ | Citation searching (sometimes called forward citation tracking), starts with a known key paper, then identifies further potential papers by collecting the references that have cited the original paper. It assumes that if the first paper is relevant then later papers that cite the original paper may also be potentially relevant. Because citation searching is not based on pre-specified terminology it has the potential to retrieve studies that are not retrieved by the keyword-based search strategies that are conducted in bibliographic databases and other resources. This makes citation searching particularly effective in systematic reviews where the search terms are difficult to define, usefully extending to iterative citation searching of citations identified by citation searching (also known as ‘snowballing’).  Citation searching can be conducted with applications such as SCOPUS, Google Scholar, and Web of Science. |
| Reference checking^2,3^ | Checking reference lists within eligible studies supplements other searching approaches and may reveal new studies or confirm that the topic has been thoroughly searched. Checking reference lists might be particularly beneficial where the intervention is one which crosses subject disciplines, for example, between health and other fields such as education, psychology, or social work. Researchers may use different terminology to describe an intervention depending on their field, making database searching difficult. |
| Outcomes measurement properties filter search^4^ | A highly sensitive search filter (developed using PubMed) for finding studies on measurement properties of measurement instruments and a more precise search filter that needs less abstracts to be screened, but at a higher risk of missing relevant studies. |

**References**

1. Wright K, Golder S, Rodriguez-Lopez R. Citation searching: a systematic review case study of multiple risk behaviour interventions. *BMC Med Res Methodol.* 2014;14(1):1-8.

2. Lefebvre C, Glanville J, Briscoe S, et al. Technical Supplement to Chapter 4: Searching for and selecting studies. *Cochrane Handbook for Systematic Reviews of Interventions Version 6.3 (updated February 2022)* 2022; [www.training.cochrane.org/handbook](https://theottawahospital.sharepoint.com/sites/SquiresTeam/Shared%20Documents/General/2020%20Context-Measurement/Terwee%20Method%20Study/Terwee%20Method%20MS%20feedback/Additional%20file/www.training.cochrane.org/handbook). Accessed August 15, 2022.

3. Horsley T, Dingwall O, Sampson M. Checking reference lists to find additional studies for systematic reviews. *Cochrane Database Syst Rev.* 2011(8).

4. Terwee CB, Jansma EP, Riphagen II, de Vet HC. Development of a methodological PubMed search filter for finding studies on measurement properties of measurement instruments. *Qual Life Res.* 2009;18(8):1115-1123.
