# Supplementary material for: Insights from using an outcomes measurement properties search filter and conducting citation searches to locate psychometric articles of tools used to measure context attributes
Source: BMC Res Notes. 2023 Mar 11;16:34. doi: 10.1186/s13104-023-06294-2 (PMC10007786; doi:10.1186/s13104-023-06294-2)
Supplement: Supplementary file 2 — Additional file 2: Tool names and search terms combined with terwee filter. [file 13104_2023_6294_MOESM2_ESM.docx]

**Additional file 2 -** Tool Names and Search Terms Combined with Terwee Filter

| **Tool Name** | **Search Terms combined with the outcomes measurement properties filter** | **Search Date** | **Outcomes measurement properties filter** | **Citation Search** | **Indexed Article Used for Citation Search** |
| --- | --- | --- | --- | --- | --- |
| American Board of Internal Medicine's (ABIM) questionnaire | American Board of Internal Medicine's questionnaire | 25-Sep-21 | Precise | No | Citation searching was not performed |
| Art of Medicine survey | Art of Medicine survey | 08-Oct-21 | Precise | No | Citation searching was not performed |
| Care Transition(s) Measure | “Care Transitions Measure” OR "Care Transition Measure" | 08-Oct-21 | Precise | No | Citation searching was not performed |
| ColloboRATE measure | "CollaboRATE" AND measure | 08-Oct-21 | Precise | No | Citation searching was not performed |
| Community Impacts of Research Oriented Partnerships (CIROP) | Community Impacts of Research Oriented Partnerships OR CIROP | 2021-12-16 | Precise | No | Citation searching was not performed |
| Doctors' Interpersonal Skills Questionnaire (DISQ) | Doctors' Interpersonal Skills Questionnaire OR DISQ | 08-Oct-21 | Precise | No | Citation searching was not performed |
| No explicit name-please see source | Conducted a citation search as unnamed | 18-Jul-21 | None | No | Citation searching was not performed |
| Global Transformational Leadership Scale | Global Transformational Leadership Scale | 2021-08-01 | Precise | Yes | Carless SA, Wearing AJ, Mann L. A short measure of transformational leadership. *J Bus Psychol*. 2000 Sep;14(3):389-405. |
| Implementation Leadership Scale* | Implementation Leadership Scale | 18-Jul-21 | Precise | No | Aarons GA, Ehrhart MG, Farahnak LR. The implementation leadership scale (ILS): development of a brief measure of unit level implementation leadership. *Implement Sci*. 2014 Dec;9(1):1-0. |
| Internal Participation Scale | Internal Participation Scale | 2021-12-16 | Precise | No | Citation searching was not performed |
| Interpersonal processes of care: IPC -29* | “Interpersonal processes of care” OR IPC -29 | 25-Sep-21 | Precise | No | Stewart AL, Nápoles‐Springer AM, Gregorich SE, Santoyo‐Olsson J. Interpersonal processes of care survey: patient‐reported measures for diverse groups. Health services research. 2007 Jun;42(3p1):1235-56. |
| Leadership Behavior Description Questionnaire | Leadership Behavior Description Questionnaire | 2021-08-01 | Precise | No | Citation searching was not performed |
| Multifactor Leadership Questionnaire (MLQ) | Multifactor Leadership Questionnaire OR MLQ | 05-Sep-21 | Precise | No | Citation searching was not performed |
| Multiple-group measurement scale for interprofessional collaboration* | multiple-group measurement scale for interprofessional collaboration | 2021-12-16 | Precise | No | Kenaszchuk C, Reeves S, Nicholas D, Zwarenstein M. Validity and reliability of a multiple-group measurement scale for interprofessional collaboration. *BMC Health Serv. Res*. 2010 Dec;10(1):1-5. |
| Patient Participation Emergency Department questionnaire (PPED) | Patient Participation Emergency Department questionnaire OR PPED | 08-Oct-21 | Precise | No | Citation searching was not performed |
| Perceived Health Web Site Usability Questionnaire (PHWSUQ) | Perceived Health Web Site Usability Questionnaire OR PHWSUQ | 2021-12-16 | Precise | No | Citation searching was not performed |
| Questionnaire on Computer Systems and Decision making | Questionnaire on Computer Systems and Decision making | 2021-12-16 | Precise | Yes | Hatcher M. Survey of acute care hospitals in the United States relative to technology usage and technology transfer. *J. Med. Syst.* 1997 Oct;21(5):323-36. |
| Risser Patient Satisfaction Scale* | Risser Patient Satisfaction Scale OR "Patient Satisfaction Scale" OR "Patient Satisfaction Instrument" OR "Patient Satisfaction Inex" | 08-Oct-21 | Precise | No | Risser N: Development of an instrument to measure patient satisfaction with nurses and nursing care in primary care settings. *Nurs Res*. 1975, 24:45–52. |
| Role Based Performance Scale | Role Based Performance Scale | 2021-09-17 | Sensitive | Yes | Welbourne TM, Johnson DE, Erez A. The role-based performance scale: Validity analysis of a theory-based measure. *Acad. Manag. J.* 1998 Oct 1;41(5):540-55. |
| SDM-Q-Doc | SDM-Q-Doc | 2021-12-16 | Precise | No | Citation searching was not performed |
| Servant Leadership Questionnaire | Servant leadership | 19-Aug-21 | Precise | Yes | Barbuto Jr JE, Wheeler DW. Scale development and construct clarification of servant leadership. *Group Organ Manag*. 2006 Jun;31(3):300-26. |
| Servant leadership Survey | Servant leadership | 19-Aug-21 | Precise | No | Citation searching was not performed |
| Shared Decision Making Questionnaire (SDM-Q-9)* | “Shared Decision Making Questionnaire” OR SDM-Q-9 | 25-Sep-21 | Precise | No | Kriston L, Scholl I, Hölzel L, Simon D, Loh A, Härter M. The 9-item shared decision making questionnaire (SDM-Q-9). Development and psychometric properties in a primary care sample. *Patient Educ Couns*. 2010;80(1):94–9 |
| Stanford University Patient Safety Climate in Healthcare Organizations questionnaire | Stanford University Patient Safety Climate in Healthcare Organizations questionnaire | 2021-12-16 | Precise | No | Citation searching was not performed |
| Survey on Patient Safety in Ambulatory Care Organizations | Survey on Patient Safety in Ambulatory Care Organizations | 2021-12-16 | Precise | No | Citation searching was not performed |
| Team Climate Inventory (TCI) * | "Team Climate Inventory short" OR “Team Climate Inventory" | 17-Dec-21 | Precise | No | Anderson NR, West MA. Measuring climate for work group innovation: development and validation of the team climate inventory. *J Occup Organ Psychol*. 1998 May;19(3):235-58. |
| The Mentorship Effectiveness Scale | The Mentorship Effectiveness Scale | 19-Aug-21 | Precise | No | Citation searching was not performed |
| The Patient Assessment of Chronic Illness Care Survey | The Patient Assessment of Chronic Illness Care Survey OR PACIC | 2021-09-17 | Precise | No | Citation searching was not performed |
| Trust in Health Promotion Partnerships Scale | Trust in Health Promotion Partnerships Scale | 2021-12-16 | Precise | No | Citation searching was not performed |
| No explicit name-please see source | Conducted a citation search | 18-Jul-21 | None | No | Citation searching was not performed |
| Urgent Care System Questionnaire (UCSQ) | Urgent Care System Questionnaire OR UCSQ | 08-Oct-21 | Precise | No | Citation searching was not performed |

Note: *The six tools included in sub analysis
