# Supplementary material for: Insights from using an outcomes measurement properties search filter and conducting citation searches to locate psychometric articles of tools used to measure context attributes
Source: BMC Res Notes. 2023 Mar 11;16:34. doi: 10.1186/s13104-023-06294-2 (PMC10007786; doi:10.1186/s13104-023-06294-2)
Supplement: Supplementary file 3 — Additional file 3: PRISMA diagram of identified records screened and full texts appraised. [file 13104_2023_6294_MOESM3_ESM.docx]

**Additional File 3** – PRISMA diagram of identified records screened and full texts appraised

**Identification of studies via databases and registers**

Records identified from*:

PubMed using the precise filter (n = 4,430)

PubMed using the sensitive filter (n = 3)

Citation searches (n = 2,134)

Reference checks of included articles (n = 4702)

Records removed *before screening*:

Duplicate records removed (n = 966)

**Identification**

Records screened

(n = 10,303)

Records excluded**

(n = 9750)

Reports sought for retrieval

(n = 553)

Reports not retrieved

(n = 7)

**Screening**

Reports excluded (n = 396):

Not psychometric (n = 211)

Previously included (n = 93)

Not English (n = 17)

Not health care (n = 28)

Reviews or qualitative studies (n = 21)

Wrong tool (n = 26)

Reports assessed for eligibility

(n = 546)

Articles identified via:

Search with Terwee precise filter (n = 130)

Reference check of included articles (n = 12)

Citation searching of development article (n = 8)

Articles included in review

(n = 150)

**Included**

*Consider, if feasible to do so, reporting the number of records identified from each database or register searched (rather than the total number across all databases/registers).

**If automation tools were used, indicate how many records were excluded by a human and how many were excluded by automation tools.

*From:*  Page MJ, McKenzie JE, Bossuyt PM, Boutron I, Hoffmann TC, Mulrow CD, et al. The PRISMA 2020 statement: an updated guideline for reporting systematic reviews. BMJ 2021;372:n71. doi: 10.1136/bmj.n71
