# Supplementary material for: Insights from using an outcomes measurement properties search filter and conducting citation searches to locate psychometric articles of tools used to measure context attributes
Source: BMC Res Notes. 2023 Mar 11;16:34. doi: 10.1186/s13104-023-06294-2 (PMC10007786; doi:10.1186/s13104-023-06294-2)
Supplement: Supplementary file 4 — Additional file 4: Search results breakdown for each tool. [file 13104_2023_6294_MOESM4_ESM.docx]

**Additional File 4:** Search Results Breakdown for Each Tool

| **Tool Details** | | **Total** | | | **Outcomes measurement properties filter** | | | **Reference check** | | | **Citation Search** | | |
| --- | --- | --- | --- | --- | --- | --- | --- | --- | --- | --- | --- | --- | --- |
| **Tool name** | **PROMS/ Non-PROMS** | **# of records** | **# of full texts** | **# of relevant articles** | **# of records** | **# of full text** | **# of relevant articles** | **# of records** | **# of full texts** | **# of relevant articles** | **# of records** | **# of full texts** | **# of relevant articles** |
| American Board of Internal Medicine's (ABIM) questionnaire | Y | 84 | 9 | 0 | 84 | 9 | 0 | N/A | N/A | N/A | 0 | 0 | 0 |
| Art of Medicine survey | Y | 703 | 0 | 0 | 703 | 0 | 0 | N/A | N/A | N/A | N/A | N/A | N/A |
| Care Transitions Measure | Y | 308 | 44 | 8 | 42 | 18 | 8 | 266 | 26 | 0 | N/A | N/A | N/A |
| ColloboRATE measure | Y | 543 | 26 | 13 | 298 | 14 | 10 | 245 | 12 | 3 | N/A | N/A | N/A |
| Community Impacts of Research Oriented Partnerships (CIROP) | N | 78 | 2 | 1 | 16 | 1 | 1 | 62 | 1 | 0 | 452 | 7 | 2 |
| Doctors' Interpersonal Skills Questionnaire (DISQ) | Y | 345 | 7 | 1 | 323 | 3 | 1 | 22 | 0 | 0 | 10 | 4 | 0 |
| No explicit name-please see source | N | 542 | 6 | 2 | N/A | N/A | N/A | 103 | 3 | 0 | 439 | 3 | 1 |
| Global Transformational Leadership Scale | N | 1 | 0 | 2 | 1 | 0 | 0 | 143 | 0 | 0 | N/A | N/A | N/A |
| Implementation Leadership Scale* | N | 471 | 22 | 9 | 185 | 10 | 9 | 286 | 12 | 0 | N/A | N/A | N/A |
| Internal Participation Scale | N | 56 | 1 | 1 | 3 | 1 | 1 | 53 | 0 | 0 | N/A | N/A | N/A |
| Interpersonal processes of care: IPC -29* | Y | 392 | 21 | 9 | 124 | 11 | 9 | 268 | 10 | 0 | N/A | N/A | N/A |
| Leadership Behavior Description Questionnaire | N | 76 | 4 | 0 | 76 | 4 | 0 | N/A | N/A | N/A | N/A | N/A | N/A |
| Multifactor Leadership Questionnaire (MLQ) | N | 184 | 30 | 9 | 68 | 8 | 4 | 116 | 22 | 5 | N/A | N/A | N/A |
| Multiple-group measurement scale for interprofessional collaboration* | N | 97 | 3 | 2 | 2 | 2 | 2 | 95 | 1 | 0 | N/A | N/A | N/A |
| Patient Participation Emergency Department questionnaire (PPED) | Y | 163 | 2 | 1 | 120 | 1 | 1 | 43 | 1 | 0 | N/A | N/A | N/A |
| Perceived Health Web Site Usability Questionnaire (PHWSUQ) | N | 45 | 5 | 2 | 5 | 3 | 2 | 40 | 2 | 0 | 76 | 10 | 1 |
| Questionnaire on Computer Systems and Decision making | N | 452 | 4 | 0 | 452 | 4 | 0 | N/A | N/A | N/A | N/A | N/A | N/A |
| Risser Patient Satisfaction Scale* | Y | 245 | 17 | 6 | 114 | 11 | 6 | 131 | 7 | 0 | N/A | N/A | N/A |
| Role Based Performance Scale | N | 58 | 0 | 1 | 3 | 0 | 0 | 55 | 0 | 0 | N/A | N/A | N/A |
| SDM-Q-Doc | Y | 196 | 23 | 7 | 14 | 9 | 7 | 182 | 14 | 0 | N/A | N/A | N/A |
| Servant Leadership Questionnaire | N | 141 | 20 | 2 | 22 | 8 | 0 | 119 | 12 | 0 | N/A | N/A | N/A |
| Servant leadership Survey | N | 205 | 24 | 4 | 22 | 8 | 3 | 183 | 16 | 1 | N/A | N/A | N/A |
| Shared Decision Making Questionnaire (SDM-Q-9)* | Y | 489 | 58 | 22 | 55 | 29 | 22 | 434 | 29 | 0 | N/A | N/A | N/A |
| Stanford University Patient Safety Climate in Healthcare Organizations questionnaire | N | 47 | 6 | 1 | 7 | 2 | 1 | 40 | 4 | 0 | N/A | N/A | N/A |
| Survey on Patient Safety in Ambulatory Care Organizations | N | 136 | 8 | 1 | 92 | 6 | 1 | 44 | 2 | 0 | N/A | N/A | N/A |
| Team Climate Inventory (TCI) * | N | 118 | 32 | 8 | 20 | 16 | 5 | 98 | 16 | 3 | N/A | N/A | N/A |
| The Mentorship Effectiveness Scale | N | 138 | 4 | 1 | 80 | 2 | 1 | 58 | 2 | 0 | N/A | N/A | N/A |
| The Patient Assessment of Chronic Illness Care Survey | Y | 984 | 81 | 35 | 479 | 42 | 34 | 505 | 39 | 1 | N/A | N/A | N/A |
| Trust in Health Promotion Partnerships Scale | N | 41 | 3 | 1 | 6 | 3 | 1 | 35 | 0 | 0 | N/A | N/A | N/A |
| No explicit name-please see source | N | 92 | 5 | 1 | N/A | N/A | N/A | 92 | 5 | 0 | N/A | N/A | N/A |
| Urgent Care System Questionnaire (UCSQ) | Y | 1035 | 2 | 1 | 1017 | 2 | 1 | 18 | 0 | 0 | 479 | 24 | 2 |
| **Total** |  | **10303** | **553** | **150** | **4433** | **227** | **130** | **3736** | **235** | **12** | **2134** | **91** | **8** |

Note: *The six tools included in sub analysis
