# Supplementary material for: Insights from using an outcomes measurement properties search filter and conducting citation searches to locate psychometric articles of tools used to measure context attributes
Source: BMC Res Notes. 2023 Mar 11;16:34. doi: 10.1186/s13104-023-06294-2 (PMC10007786; doi:10.1186/s13104-023-06294-2)
Supplement: Supplementary file 5 — Additional file 5: Reasons for discrepancies between the precise outcomes measurement properties filter search and citation sear. [file 13104_2023_6294_MOESM5_ESM.docx]

**Additional file 5:** Reasons for Discrepancies Between the Precise Outcomes Measurement Properties Filter Search and Citation Search

**1. Tool Name:** **Interpersonal Processes of Care**

**Article Used for Citation Search**: Stewart AL, Nápoles‐Springer AM, Gregorich SE, Santoyo‐Olsson J. Interpersonal processes of care survey: patient‐reported measures for diverse groups. Health services research. 2007 Jun;42(3p1):1235-56.

| **Outcomes Measurement Properties Filter** | |
| --- | --- |
| **Articles found with the precise filter and/or reference checks but not with Citation Searching** | **Reason** |
| Beaulieu MD, Haggerty JL, Beaulieu C, Bouharaoui F, Lévesque JF, Pineault R, Burge F, Santor DA. Interpersonal communication from the patient perspective: comparison of primary healthcare evaluation instruments. Healthcare Policy. 2011 Dec;7(Spec Issue):108. | Cites  Stewart AL, Nápoles‐Springer A, Pérez‐Stable EJ. Interpersonal processes of care in diverse populations. The Milbank Quarterly. 1999 Sep;77(3):305-39. |
| Haggerty JL, Beaulieu C, Lawson B, Santor DA, Fournier M, Burge F. What patients tell us about primary healthcare evaluation instruments: response formats, bad questions and missing pieces. Healthcare Policy. 2011 Dec;7(Spec Issue):66. | Cites  Stewart AL, Nápoles‐Springer A, Pérez‐Stable EJ. Interpersonal processes of care in diverse populations. The Milbank Quarterly. 1999 Sep;77(3):305-39. |
| Nápoles-Springer AM, Santoyo-Olsson J, O'Brien H, Stewart AL. Using cognitive interviews to develop surveys in diverse populations. Medical care. 2006 Nov 1:S21-30. | Cites  Stewart AL, Nápoles‐Springer A, Pérez‐Stable EJ. Interpersonal processes of care in diverse populations. The Milbank Quarterly. 1999 Sep;77(3):305-39. |
| Stewart AL, Nápoles‐Springer A, Pérez‐Stable EJ. Interpersonal processes of care in diverse populations. The Milbank Quarterly. 1999 Sep;77(3):305-39. | This article was published before the article that was used for the citation search. |
| **Citation Search** | |
| **Articles found Citation Searching but not with precise filter** | **Reason** |
| None | None |

2**. Tool Name:** Risser Patient Satisfaction Scale/Instrument

**Article Used for Citation Searching**: Risser N: Development of an instrument to measure patient satisfaction with nurses and nursing care in primary care settings. *Nurs Res*. 1975, 24:45–52.

| **Terwee Filter** | |
| --- | --- |
| **Articles found with the precise filter and/or reference checks but not with Citation Searching** | **Reason** |
| None | None |
| **Citation Search** | |
| **Articles found Citation Searching but not with precise filter** | **Reason** |
| None | None |

**3. Tool Name:** Shared Decision-Making Questionnaire (SDM-Q-9)

**Article Used for Citation Searching**: Kriston L, Scholl I, Hölzel L, Simon D, Loh A, Härter M. The 9-item shared decision-making questionnaire (SDM-Q-9). Development and psychometric properties in a primary care sample. *Patient Educ Couns*. 2010;80(1):94–9.

| **Terwee Filter** | |
| --- | --- |
| **Articles found with the precise filter and/or reference checks but not with Citation Searching** | **Reason** |
| Kasper J, Heesen C, Köpke S, Fulcher G, Geiger F. Patients' and observers' perceptions of involvement differ. Validation study on inter-relating measures for shared decision making. PloS one. 2011 Oct 17;6(10): e26255. | Article cites a different article:  Simon D, Schorr G, Wirtz M (2006) Development and first validation of the shared decision-making questionnaire (SDM-Q). Patient Educ Couns 63: 319–327 |
| Goto Y, Miura H, Son D, Arai H, Kriston L, Scholl I, Härter M, Sato K, Kusaba T. Psychometric evaluation of the Japanese 9-item shared decision-making questionnaire and its association with decision conflict and patient factors in Japanese primary care. JMA journal. 2020 Jul 15;3(3):208-15. | Cites the article used for the citation search but it is not indexed on Scopus or Web of Science as one of the “cited” articles. |
| **Citation Search** | |
| **Articles found Citation Searching but not with the precise filter** | **Reason** |
| None | None |

**4. Tool Name:** Implementation Leadership Scale

**Article Used for Citation Searching**: Aarons GA, Ehrhart MG, Farahnak LR. The implementation leadership scale (ILS): development of a brief measure of unit level implementation leadership. *Implement Sci*. 2014 Dec;9(1):1-0.

| **Terwee Filter** | |
| --- | --- |
| **Articles found with the precise filter and/or reference checks but not with Citation Searching** | **Reason** |
| Lyon AR, Cook CR, Brown EC, Locke J, Davis C, Ehrhart M, Aarons GA. Assessing organizational implementation context in the education sector: confirmatory factor analysis of measures of implementation leadership, climate, and citizenship. *Implement Sci. 2018* Dec;13(1):1-4. | Article cites a different article:  Finn NK, Torres EM, Ehrhart MG, Roesch SC, Aarons GA. Cross-validation of the Implementation Leadership Scale (ILS) in child welfare service organizations. Child Maltreat. 2016; 21:250–5 |
| **Citation Search** | |
| **Articles found Citation Searching but not with precise filter** | **Reason** |
| None | None |

**5. Tool Name:** Multiple-group measurement scale for interprofessional collaboration

**Article Used for Citation Searching**: Kenaszchuk C, Reeves S, Nicholas D, Zwarenstein M. Validity and reliability of a multiple-group measurement scale for interprofessional collaboration. *BMC Health Serv. Res*. 2010 Dec;10(1):1-5.

| **Terwee Filter** | |
| --- | --- |
| **Articles found with the precise filter and/or reference checks but not with Citation Searching** | **Reason** |
| None | None |
| **Citation Search** | |
| **Articles found Citation Searching but not with precise filter** | **Reason** |
| None | None |

**6. Tool Name:** Team Climate Inventory (TCI) and Team Climate Inventory-Short

**Article Used for Citation Searching**: Anderson NR, West MA. Measuring climate for work group innovation: development and validation of the team climate inventory. *J Occup Organ Psychol*. 1998 May;19(3):235-58.

| **Terwee Filter** | |
| --- | --- |
| **Articles found with the precise filter and/or reference checks but not with Citation Searching** | **Reason** |
| Kivimäki M, Kuk G, Elovainio M, Thomson L, Kalliomäki‐Levanto T, Heikkilä A. The Team Climate Inventory (TCI)—four or five factors? Testing the structure of TCI in samples of low and high complexity jobs. Journal of Occupational and Organizational Psychology. 1997 Dec;70(4):375-89. | This paper cites the article used for the citation search, but it was not captured because it is not indexed in Scopus. It is indexed in Web of Science*,* but it is not indexed as a “cited” article for the article used for the citation search. |
| Anderson N, West MA. The Team Climate Inventory: Development of the TCI and its applications in teambuilding for innovativeness. European Journal of work and organizational psychology. 1996 Mar 1;5(1):53-66. | This article was published before the article that was used for the citation search. A citation search using this paper was not completed as it is not indexed in Web of Science and Scopus. |
| **Citation Search** | |
| **Articles found Citation Searching but not with precise filter** | **Reason** |
| Antino M, Gil-Rodriguez F, Martí M, Barrasa A, Borzillo S. Development and validation of the Spanish version of the Team Climate Inventory: a measurement invariance test. Anales de Psicología/Annals of Psychology. 2014 Apr 7;30(2):597-607. | Not indexed in PubMed |
| Tseng HM, Liu FC, West MA. The team climate inventory (TCI) a psychometric test on a Taiwanese sample of work groups. Small Group Research. 2009 Aug;40(4):465-82. | Not indexed in PubMed |
