# Supplementary material for: Insights from using an outcomes measurement properties search filter and conducting citation searches to locate psychometric articles of tools used to measure context attributes
Source: BMC Res Notes. 2023 Mar 11;16:34. doi: 10.1186/s13104-023-06294-2 (PMC10007786; doi:10.1186/s13104-023-06294-2)
Supplement: Supplementary file 6 — Additional file 6: Sub-group analysis of terwee filter (with or without reference checking) and citation search. [file 13104_2023_6294_MOESM6_ESM.docx]

**Additional File 6**: Sub-group Analysis of Terwee filter (with or without reference checking) and Citation Search

| **Tool Name** | **Search Method** | **# of unique records identified** | **# of potentially relevant articles** | **# of included psychometric articles** | **Precision^1^**  **N (%)** | **Sensitivity^2^**  **N (%)** |
| --- | --- | --- | --- | --- | --- | --- |
| **Patient Reported Outcomes (PROM)** | | | | | | |
| **Interpersonal Processes of Care (PROM)** | Precise filter search and reference checks | 392 | 21 | 9 | 9/392 (2.3%) | 9/9 (100.0%) |
|  | Precise filter alone | 124 | 11 | 9 | 9/124 (7.3%) | 9/9 (100.0%) |
|  | Citation search* | 285 | 12 | 9 | 9/285 (3.2%) | 9/9 (100.0%) |
| **Risser Patient Satisfaction Scale/Instrument (PROM)** | Precise filter search and reference checks | 245 | 17 | 6 | 6/245 (2.4%) | 6/6 (100.0%) |
|  | Precise filter alone | 114 | 11 | 6 | 6/114 (5.3%) | 6/6 (100.0%) |
|  | Citation search | 278 | 126 | 6 | 6/278 (2.2%) | 6/6 (100.0%) |
| **Shared Decision-Making Questionnaire (SDM-Q-9) (PROM)** | Precise filter search and reference checks | 489 | 59 | 22 | 22/489 (4.5%) | 22/22 (100.0%) |
|  | Precise filter alone | 55 | 31 | 22 | 22/55 (40.0%) | 22/22 (100.0%) |
|  | Citation search* | 483 | 30 | 21 | 21/483 (4.3%) | 21/22 (95.4%) |
| **Subtotal** | Precise filter search and reference checks | 1126 | 97 | 37 | 31/1126 (2.8%) | Mean (SD) = 100.0% (0.0%) |
|  | Precise filter alone | 293 | 53 | 37 | 37/293 (12.6%) | Mean (SD) =  100.0% (0.0%) |
|  | Citation search | 1046 | 168 | 36 | 36/1046 (3.4%) | Mean (SD) =  98.5% (2.7%) |
| **Non-Patient Reported Outcomes (non-PROM)** | | | | | | |
| **Implementation Leadership Scale (nonPROM)** | Precise filter search and reference checks | 471 | 22 | 9 | 9/471 (1.9%) | 9/9 (100.0%) |
|  | Precise filter alone | 185 | 10 | 9 | 9/185 (4.9%) | 9/9 (100.0 %) |
|  | Citation search | 194 | 22 | 8 | 8/194 (4.1%) | 8/9 (88.9%) |
| **Multiple-group measurement scale for interprofessional collaboration (nonPROM)** | Precise filter search and reference checks | 97 | 3 | 2 | 2/97 (2.1%) | 2/2 (100.0%) |
|  | Precise filter alone | 2 | 2 | 2 | 2/2 (100.0%) | 2/2 (100.0%) |
|  | Citation search | 69 | 12 | 2 | 2/69 (2.9%) | 2/2 (100.0%) |
| **Team Climate Inventory (TCI) and Team Climate Inventory-Short (nonPROM)** | Precise filter search and reference checks | 118 | 32 | 8 | 8/118 (6.8%) | 8/10 (80.0%) |
|  | Precise filter alone | 20 | 16 | 6 | 6/20 (30.0%) | 6/10 (60.0%) |
|  | Citation search | 1226 | 28 | 8 | 8/1226 (0.7%) | 8/10 (80.0%) |
| **Subtotal for PROM** | Precise filter search and reference checks | 756 | 57 | 19 | 19/756 (2.5%) | Mean (SD) =  93.3% (11.5%) |
|  | Precise filter alone | 207 | 28 | 17 | 17/207 (8.2%) | Mean (SD) = 86.7% (23.1%) |
|  | Citation search | 1489 | 62 | 18 | 18/1489 (1.2%) | Mean (SD) = 89.6% (10.0%) |
| **Total** | **Precise filter search and reference checks** | **1882** | **154** | **56** | **56/1882 (3.0%)** | **Mean (SD) = 96.7% (8.2%)** |
|  | **Precise filter alone** | **500** | **81** | **54** | **54/500 (10.8%)** | **Mean (SD) = 93.3% (16.3%)** |
|  | **Citation search** | **2535** | **230** | **54** | **54/2535 (2.1%)** | **Mean (SD) = 94.1% (8.1%)** |

*Citation search results that changed when additional citation searches were performed. **^1^**Precision was calculated by taking the number of relevant psychometric articles included after full text screening divided by the total number of reports identified through the specific search method (i.e., searching with the precise filter or the citation search of the tool’s development article).
